# A coincidence‐based response matrix for correction of charge sharing spectral distortions in photon counting detectors

**DOI:** 10.1002/mp.70518

**Published:** 2026-06-08

**Authors:** Vincenzo Monaco, Luca Brombal, Pasquale Delogu, Alessandro Feruglio, Massimiliano Fiorini, Renata Longo, Luca Marchetti, Anna Maria Poli, Luigi Rigon, Valeria Rosso

**Affiliations:** ^1^ Dipartimento di Fisica Università degli Studi di Torino Torino Italy; ^2^ Istituto Nazionale di Fisica Nucleare Sezione di Torino Torino Italy; ^3^ Dipartimento di Fisica Università degli Studi di Trieste Trieste Italy; ^4^ Istituto Nazionale di Fisica Nucleare Sezione di Trieste Trieste Italy; ^5^ Dipartimento di Fisica Università di Pisa Pisa Italy; ^6^ Istituto Nazionale di Fisica Nucleare Sezione di Pisa Pisa Italy; ^7^ Dipartimento di Fisica e Scienze della Terra Università degli Studi di Ferrara Ferrara Italy; ^8^ Istituto Nazionale di Fisica Nucleare Sezione di Ferrara Ferrara Italy; ^9^ Technical University of Munich, Chair of Biomedical Physics Garching Germany

**Keywords:** charge sharing corrections, photon counting, X‐ray spectral imaging

## Abstract

**Background:**

Charge sharing between pixels distorts the count and spectral information of X‐ray photon counting detectors. Compensation methods for charge sharing effects are required to exploit the full potentiality of these detectors in medical diagnosis.

**Purpose:**

A statistical method is proposed to correct charge sharing effects in a pixellated photon counting detector by applying a spectral response matrix determined with a coincidence‐based acquisition.

**Methods:**

The technique is based on a preliminary calibration with a uniform irradiation and an arbitrary polychromatic spectrum, during which the number of coincidences between a pixel and its eight neighbours are collected for different combinations of energy bins. A coincidence‐based response matrix (CBRM) is determined and afterwards applied to correct other spectra acquired with the same detector and conventional multi‐comparator electronics. The technique was validated with Geant4 Monte Carlo simulations of a 1 mm thick CdTe detector and with data collected with a pixel hybrid detector consisting of a 300 μm thick silicon sensor readout by a Timepix4 chip. The effect of pulse pileup was not analyzed in this study.

**Results:**

The response matrix restores the spectral information with a performance comparable to analog charge summing (ACS) algorithms. For example, for a simulation of a spectrum from a 120 kV X‐ray tube attenuated by a solution of water and iodine and a CdTe detector with a pixel size of 200 *µ*m, the mean absolute percentage errors (MAPE) from the comparison of the corrections with an ideal spectrum are 20.0% for the CBRM method and 22.8% for ACS. The ACS method is more sensitive to electronic noise than the CBRM correction, thus requiring a higher noise discrimination threshold. For experimental acquisitions of monochromatic spectra with the silicon sensor, the mean values and standard deviations of Gaussian fits of the restored energy peaks provide results close to those from a clustering algorithm based on 3 × 3 pixel blocks. The MAPE value from the comparison of the CBRM correction and clustering distributions for a polychromatic spectrum of an X‐ray tube at 50 kV attenuated by an Ag solution is 6.2% It is also shown that a CBRM matrix determined with a fine division of the energy range can be adapted to match a lower number of energy bins employed for subsequent acquisitions without affecting the accuracy of the spectrum correction. A preliminary reconstruction of a nonuniform irradiation demonstrates the potentiality of the method to restore general spectral images.

**Conclusions:**

The proposed method allows the experimental determination of a response matrix which is independent of physics models or parameterizations and is realizable with a simple coincidence electronic circuit involving a limited number of pixels in a calibration stage. With respect to ACS techniques, the application of the response matrix requires only the number of counts collected with existing readout systems with multiple comparators, without introducing additional dead‐times during the acquisition.

## 1 INTRODUCTION

Photon counting detectors (PCDs) are rapidly emerging as a new technology for X‐ray imaging in medical diagnostics^1^, ^2^, ^3^ Planar semiconductors, mainly high‐Z cadmium‐telluride (CdTe) and cadmium‐zinc‐telluride (CZT), provide a direct conversion of the released energy in a measurable charge.^4^ When coupled to electronics with pulse‐height capabilities, it is possible to detect each interacting photon and measure its energy.

In X‐ray Computed Tomography, PCDs have demonstrated suppression of electronic noise, higher spatial resolution, reduced dose for the same image quality, higher dynamic range, multi‐material decomposition and enhanced contrast agent discrimination with respect to traditional charge‐integrating detectors.^5^ A first commercial scanner for X‐ray photon counting Computed Tomography was introduced by Siemens Healthineers in 2021, confirming the clinical benefits of this technology.^6^, ^7^

Despite their intrinsic advantages, the performance of PCDs in spectroscopic X‐ray imaging is limited by two deleterious effects: the sharing of the signals between neighbouring pixels^8^, ^9^ and the overlap of pulses from events occurring too close in time (pulse pileup, PPU).^10^, ^11^ Both these effects distort the spectral distribution of counts in each pixel and degrade the information obtainable from the reconstructed images.^12^ PPU distortions are particularly critical in clinical photon counting Computed Tomography, where the X‐ray flux could be of the order of $10^{9}$ $s^{-1}{\mathrm{mm}}^{-2}$.

The sharing of the charge between different pixels is caused by the induction of signals on two or more nearby pixels while the carriers' charges migrate toward the electrodes.^13^ This effect depends on the size of the charge cloud and on the superposition of the weighting potentials of adjacent pixels. It becomes significant in high spatial resolution applications, where the pixel pitch is small with respect to the sensor thickness. Other spectrum distortions in high‐Z sensors arise from the emission of characteristic fluorescence photons or Compton photons which can interact in other detector pixels or exit from the detector. In the following, we use a wide definition of charge sharing (CS) which includes all the mechanisms that could generate the splitting of the charge produced by an interacting photon between different pixels, giving rise to multiple hits with lower charge. Charge sharing distortions can be attenuated by using pixels of larger dimensions, but this comes at the cost of both reduced spatial resolution and a worsening of PPU effects which, on the contrary, are reduced with smaller pixels.

The readout electronics has a key role in reducing or compensating counting and spectral distortions caused by CS and PPU effects. The readout chips developed for high‐flux applications^14^, ^15^, ^16^, ^17^ employ fast shaping with short peaking times, thus achieving dead‐times of only a few tens of nanoseconds to minimize the effect of PPU, although this does not limit CS effects for small pixel sizes. These electronics are equipped with multiple comparators at different thresholds to count the number of photons releasing energies above the corresponding threshold values.

A powerful technique for CS compensation is based on the on‐line clustering of the energy measured by nearby pixels.^18^, ^19^ A dedicated analog processing is adopted to sum the energies released in groups of pixels and inter‐pixel comparators are employed to assign the resulting value to the pixel with the largest energy release. These analog charge summing (ACS) algorithms, already implemented in the electronics of the Medipix family^20^ and in other readout circuits,^21^, ^22^, ^23^, ^24^ are very effective in compensating CS effects, but introduce an additional dead‐time, which worsens the effect of PPU at high input rates. Therefore, these algorithms are not suitable for high‐flux applications.

Other approaches for the correction of PPU and/or CS effects are based on the post‐processing of the acquired spectral images. Most of the proposed analytical methods make use of semi‐empirical response functions based on a model of the processes producing spectral distortions, with parameters determined from simulations or data fitting.^25^, ^26^, ^27^ In most of these studies the response function is defined for a particular irradiation setup and detector configuration, and it is not guaranteed to be of general use in image reconstructions with different conditions.

In recent years, multi‐energy inter‐pixel coincidence counters (MEICC) were proposed for CS corrections.^28^ This method requires a dedicated readout electronics to count the number of coincidences between different energy bins for each pixel and its neighbours. Many coincidence gates and counters (of the order of $N^{2}$ per pixel where N is the number of energy bins) are needed. Post‐processing algorithms of the data collected with MEICC counters were proposed,^29^, ^30^ showing the promising potentiality of the method for compensation of CS effects.

In this paper a statistical post‐processing method for the characterization and correction of CS distortions of PCD X‐ray spectra is proposed. The method is based on the collection of calibration data with an arbitrary polychromatic spectrum and a uniform illumination of the detector to determine the elements of a spectral response matrix. At this stage it is required that the number of coincidences between the signal of one pixel and the analog sum of its neighbouring pixels are counted for different combinations of energy bins. Assuming an equalized behaviour of all the PCD pixels, the calibration requires two programmable thresholds and a simple coincidence circuit involving only a limited number of pixels. The calibration allows to define a set of probabilities on how the initial energy is split with different fractions between one pixel and its neighbours. These probabilities are used to define the elements of a matrix, here named “coincidence‐based response matrix” (CBRM), which inversion allows to retrieve the input energy distribution in the absence of CS. The CBRM is independent of the input spectrum and can therefore be used to correct CS distortions in subsequent acquisitions with different spectra. The correction requires only the integral number of counts for different energy bins collected by conventional multi‐discriminator electronics. Therefore, once the response matrix is determined, it is adapted to match the energy bins defined by the thresholds employed in a standard readout and applied to restore the input spectrum for each pixel. Compared to other semi‐empirical post‐processing algorithms, the method is based only on data inputs, is independent of physics models or simulations, is simple and of general applicability. No additional dead‐time is introduced during the acquisitions, with a potential enhancement of the acquisition efficiency with respect to ACS techniques.

The method was validated with Monte Carlo simulations of a CdTe detector and with data acquired with a silicon pixel sensor coupled with the Timepix4^31^ readout electronics using monochromatic and polychromatic X‐ray beams. Spectral distortions due to PPU were not investigated in this study.

## 2 METHODS

### 2.1 Data acquisition system

In most of the electronics for spectroscopic photon counting the signal from each pixel, after an amplification and shaping stage, is fed to a multiple‐comparator system comprising a bank of discriminators with different thresholds. Each comparator (henceforth identified by an index i) is associated to a counter register, which is incremented every time the leading edge of the signal crosses the corresponding threshold. In the following, we assume that the thresholds are expressed in terms of energy values. We indicate with $N_{i}$ the number of events for which the energy released by a photon in one pixel is above the threshold i and with $N_{i}^{\prime }$ the number of raw counts for counter i. In general, the two values are different due to CS, PPU, electronic noise, detector resolution and other effects. If PPU effects are negligible, the number of raw counts $n_{i}^{\prime }$ in the energy bin i is given by the difference of raw counts $N_{i}^{\prime }$ of adjacent thresholds.

**FIGURE 1:**
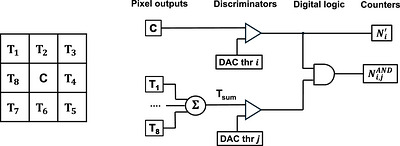
Scheme of the acquisition system used to store the information needed to determine the elements of the spectral response function. The signals from a reference pixel (C) and the sum of the analog signals from the eight surrounding pixels ($T_{sum}$) are compared against a pair of energy thresholds i and j. The number of output pulses for the reference pixels and for the logical AND of the outputs of the two discriminators are stored in counters. The procedure is repeated for all the possible combinations of threshold values.

In this study, the response matrix is determined by counting the number of coincidences between one pixel and its eight neighbours. The labelling defined in Figure 1 is used, where C is the pixel under study (in the following called reference pixel), and the symbols $T_{r}$ (with r=1,…8) identify the eight pixels surrounding the reference one. The proposed readout scheme for the calibration phase is shown in the same figure. The analog sum of the signals from the peripheral pixels ($T_{\mathrm{sum}}$) and the signal from pixel C are discriminated against two programmable thresholds. The threshold values are selected through two Digital‐to‐Analog Converters (DAC) from a set of values defining a fine division of the energy range in a number of bins. Minimum discrimination thresholds ($thr_{\mathrm{pxl}}$ for the C pixel and $thr_{\mathrm{sum}}$ for $T_{\mathrm{sum}}$) are applied to discard fake counts due to noise.

For each pair of possible thresholds i and j, the number of counts from the reference pixel $N_{i}^{\prime }$ and from the logical AND combination of the two discriminator outputs $N_{i,j}^{AND}$ are saved in counters. If PPU effects are negligible, the relation between the number of coincident events $n_{i,j}^{AND}$ in two energy bins (i,j) can be determined by combining the number of coincidences above adjacent thresholds.

The calibration is performed with a uniform irradiation of the detector and different acquisitions for all the possible combinations of the two threshold levels. Assuming a detector with equalized pixel outputs, the calibration can be performed using only one coincidence logic involving a single group of 8+1 pixels.

### 2.2 Determination and application of the CBRM matrix

#### 2.2.1 Statistical description of charge sharing effects

For the determination of the response matrix we assume that the useful energy range is divided into a large number L of energy bins of equal width ΔE. In the following we will indicate with an index i a count or an event in the energy bin between iΔE and (i+1)ΔE. We focus on a given pixel of the detector, identified as the reference pixel C of Figure 1. The statistical model used in this study is based on the assumptions listed below.

**FIGURE 2:**
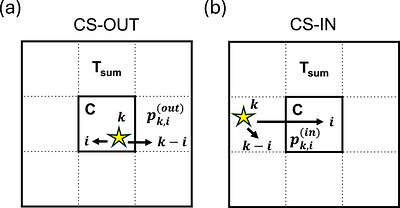
Schematic of the transition probabilities used in the CS statistical model. (a) If the interaction occurs in the reference pixel C with input energy at bin k, the released energy can be split into a signal at bin i in the reference pixel and a signal at bin k−i distributed among the surrounding eight pixels with probability $p_{k,i}^{\left(out\right)}$ (CS‐OUT transitions). (b) In a CS‐IN transition the interaction event occurs in one of the eight pixels around the reference one, and the deposited energy at bin k is split into a signal at bin i of the reference pixel and a signal at bin k−i of the peripheral pixels with probability $p_{k,i}^{\left(in\right)}$.

- When the incoming photon interacts in a pixel, the released energy can be shared only with the eight surrounding pixels.
- The charge sharing is modelled by a set of probabilities $p_{k,i}^{\left(out\right)}$ that an event in the reference pixel C with input energy at bin k is split into multiple lower energy hits, with energy in the bin i for the reference pixel and with an energy at bin k−i for the sum of the eight surrounding pixels. We refer to these events as CS‐OUT transitions (Figure 2a).
- If the interaction occurs in one of the eight pixels $T_{r}$ around C with deposited energy at bin k, the probability that a count at bin i is detected in the reference pixel is $p_{k,i}^{\left(in\right)}$. These events are named CS‐IN transitions (Figure 2b). It is also assumed that, when an interaction occurs in a peripheral pixel and a count is detected in the reference pixel, the cluster of pixels where the released energy is split does not extend outside the 3 × 3 block defined in Figure 2. The residual energy k−i is therefore collected by the eight peripheral pixels.

Charge sharing produces a distortion in the measured number of counts in energy bin i of a reference pixel due to the three processes listed below.

1. The energy released by a photon interaction in the reference pixel with input energy at bin k (with k>i) is shared with the nearby pixels reducing the energy measured in the reference pixel to bin i and adding a count to this energy bin. The probability of this CS‐OUT transition is $p_{k,i}^{\left(out\right)}$, where k−i is the residual energy in the peripheral pixels.
2. An event in the reference pixel with deposited energy at bin i is shared with the neighbouring pixels, reducing the number of counts at bin i. This CS‐OUT transition has probability $p_{i,j}^{\left(out\right)}$, where j<i is the residual energy in the reference pixel and i−j is the energy released in the nearby pixels.
3. An interaction event occurring in one of the eight neighbouring pixels with energy at bin k (with k≥i) is shared with the reference pixel, adding a count at bin i of the reference pixel and reducing the energy of the peripheral pixels to k−i. The probability of this CS‐IN transition is $p_{k,i}^{\left(in\right)}$.

For a number L of energy bins, the spectral distortion due to CS can be described in terms of transition probabilities by the following equation:

(1) $$\begin{array}{cc} n_{i}^{{\left(c\right)}^{\prime }} & =n_{i}^{\left(c\right)}+\sum_{k=i+1}^{L-1}p_{k,i}^{\left(out\right)}n_{k}^{\left(c\right)}-\sum_{j=0}^{i-1}p_{i,j}^{\left(out\right)}n_{i}^{\left(c\right)}\\ & \;+\sum_{k=i}^{L-1}p_{k,i}^{\left(in\right)}\sum_{r=1}^{8}n_{k}^{\left(t_{r}\right)} \end{array}$$

where $n_{i}^{{\left(c\right)}^{\prime }}$ is the number of raw counts at energy bin i of the reference pixel C, $n_{k}^{\left(c\right)}$ and $n_{k}^{\left(t_{r}\right)}$ are the number of input events at energy bin k respectively for the pixel C and for each of the eight surrounding pixels $T_{r}$. The three last terms in Equation (1) correspond to the three possible distortions of the number of counts at bin i described above.

The total CS‐OUT probability that an event in the reference pixel C with initial energy i is shared with the surrounding pixels is:

(2) $$PT_{i}^{\left(out\right)}=\sum_{j=0}^{i}p_{i,j}^{\left(out\right)}$$

Similarly, the total CS‐IN probability that an event in one peripheral pixel with initial energy i shares a fraction of its energy with the reference pixel is:

(3) $$PT_{i}^{\left(in\right)}=\sum_{j=0}^{i}p_{i,j}^{\left(in\right)}$$

Under the assumptions of the model, the total CS‐IN charge sharing probability is one‐eighth of the total CS‐OUT probability for the same initial energy i:

(4) $$PT_{i}^{\left(in\right)}=\frac{1}{8}PT_{i}^{\left(out\right)}$$

#### 2.2.2 CBRM matrix and spectral correction

The acquisition system of Figure 1 is used to collect the number of raw counts 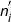 for one pixel and of coincidences $n_{i,j}^{AND}$ between this pixel and its eight neighbours, during a uniform illumination of the whole detector with a polychromatic X‐ray beam. The number of coincidences for a pair of energy bins can be expressed in terms of the CS‐OUT and CS‐IN transition probabilities as:

(5) $$n_{i,j}^{AND}=p_{i+j,i}^{\left(out\right)}n_{i+j}^{\left(c\right)}+p_{i+j,i}^{\left(in\right)}\sum_{r=1}^{8}n_{i+j}^{\left(t_{r}\right)}$$

For a uniform illumination, all the pixels have the same energy distribution, and therefore:

(6) $$n_{i}^{\left(t_{r}\right)}=n_{i}^{\left(c\right)}=n_{i}$$

where $n_{i}$ is the number of input events at energy bin i, equal for all the pixels.

Equation (5) for a uniform irradiation becomes:

(7) $$n_{i,j}^{AND}=\left(p_{i+j,i}^{\left(out\right)}+8p_{i+j,i}^{\left(in\right)}\right)n_{i+j}$$

The CS splitting probabilities are defined as:

(8) $$q_{k,i}=\left(p_{k,i}^{\left(out\right)}+8p_{k,i}^{\left(in\right)}\right)=\frac{n_{i,k-i}^{AND}}{n_{k}}$$

Equation (1) for a uniform illumination can be expressed in terms of splitting probabilities as:

(9) $$n_{i}^{\prime }=n_{i}+\sum_{k=i+1}^{L-1}q_{k,i}n_{k}-PT_{i}^{\left(out\right)}n_{i}+q_{i,i}n_{i}$$

Taking into account Equations (2), (3) and (4), we have:

(10) $$\begin{array}{cc} PT_{i}^{\left(out\right)} & =\frac{1}{2}\left(PT_{i}^{\left(out\right)}+8PT_{i}^{\left(in\right)}\right)=\frac{1}{2}\sum_{j=0}^{i}q_{i,j}\\ & =\frac{1}{2n_{i}}\sum_{j=0}^{i}n_{j,i-j}^{AND}=\frac{1}{2n_{i}}\sum_{j=0}^{i}n_{i-j,j}^{AND} \end{array}$$

and therefore Equation (9) becomes:

(11) $$n_{i}^{\prime }=n_{i}+\sum_{k=i+1}^{L-1}n_{i,k-i}^{AND}-\frac{1}{2}\sum_{j=0}^{i}n_{i-j,j}^{AND}+n_{i,0}^{AND}$$

Equation (11) allows to determine the number of input events $n_{i}$ from the number of raw counts $n_{i}^{\prime }$ and of coincidences collected in the calibration run. Therefore, the splitting probabilities $q_{k,j}$ can be determined from Equation (8) and applied to compensate CS distortions for different spectra acquired with a readout based on a bank of discriminators and counters. If the set of thresholds is the same employed in the calibration, the number of input events $n_{i}$ can be retrieved from the number of raw counts $n_{i}^{\prime }$ by solving Equation (9) which, for a uniform illumination of the whole detector, becomes:

(12) $$n_{i}^{\prime }=\left[1+q_{i,i}-\frac{1}{2}\sum_{j=0}^{i}q_{i,j}\right]n_{i}+\sum_{j=i+1}^{L-1}q_{j,i}n_{j}=\sum_{j=0}^{L-1}A_{i,j}n_{j}$$

The factors $A_{i,j}$ in Equation (12) are the elements of the CBRM matrix, given by:

(13) $$A_{i,j}=\left[1+q_{i,i}-\frac{1}{2}\sum_{j^{\prime }=0}^{i}q_{i,j^{\prime }}\right]\delta_{i,j}+q_{j,i}H\left(j-i\right)$$

where $\delta_{i,j}$ is the Kronecker delta function and H(j−i) the Heaviside function (equal to 1 for j>i and 0 otherwise).

The matrix is triangular and can be easily inverted to retrieve the original number of events in each energy bin. In practice, Equation (12) can be solved in an iterative way, starting from the highest energy bin and proceeding toward lower energy bins. The solution for the number of input events at bin i is:

(14) $$n_{i}=\frac{n_{i}^{\prime }-\sum_{j=i+1}^{L-1}q_{j,i}n_{j}}{1+q_{i,i}-\frac{1}{2}\sum_{j=0}^{i}q_{i,j}}$$

#### 2.2.3 Reduction of the CBRM matrix

The existing electronics include a relatively small number of discriminators, in general lower than the number of energy bins needed for an accurate definition of the response matrix. Therefore, the splitting probabilities determined in the calibration run must be adapted to match the number and the widths of the energy bins of a multi‐comparator readout.

In the following we assume that a large number 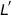 of energy bins of equal width 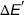 are employed for the calibration run, while in a standard acquisition the energy range is segmented into a lower number L of bins, not necessarily of the same width, defined by the thresholds $E_{i}$ of the comparators. We indicate with the index 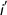 a bin for the calibration acquisition corresponding to the energy interval 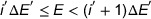, and with the index i an energy bin for the standard acquisition in the range $E_{i}\leq E<E_{i+1}$ and of width $\Delta E_{i}=E_{i+1}-E_{i}$.

For the wide energy bins defined in the standard acquisition the hypothesis that the sum of the average energies at bins i and j in the reference pixel and in the eight neighbouring pixels is equal to the average energy at bin i+j in general does not hold. The spectral distortions for a low number of energy bins are thus parameterized in terms of three‐fold CS‐OUT probabilities $P_{k,i,j}^{\left(out,in\right)}$ that an event with initial energy k released in the reference pixel (for $P_{k,i,j}^{\left(out\right)}$) or in one of the neighbouring pixels (for $P_{k,i,j}^{\left(in\right)}$) is split into a count at bin i in the C pixel and a count at bin j in the eight surrounding pixels, with k not necessarily equal to i+j. If the set of thresholds employed in the standard acquisition is the same of the calibration run the three‐fold probabilities coincide with those defined in the calibration ($P_{k,i,j}^{\left(out,in\right)}=p_{k,i}^{\left(out,in\right)}$, with k=i+j). The probabilities $P_{k,i,j}^{\left(out\right)}$ and $P_{k,i,j}^{\left(in\right)}$ are related to the CS‐IN and CS‐OUT transition probabilities 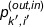 defined for the calibration run by:

(15)

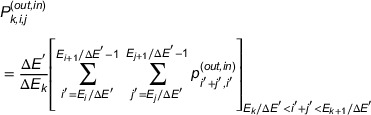




The factor multiplying the sums in Equation (15) takes into account the different number of counts in the bins of the two acquisitions to which the transition probabilities are applied.

The CS‐OUT and CS‐IN probabilities that an event with initial energy k produces a count at energy i in the reference pixel, regardless the energy shared with other pixels, are given by:

(16) $$P_{k,i}^{\left(out,in\right)}=\sum_{j=0}^{k}P_{k,i,j}^{\left(out,in\right)}$$

The splitting probabilities are defined by a combination of CS‐IN and CS‐OUT transition probabilities as:

(17)

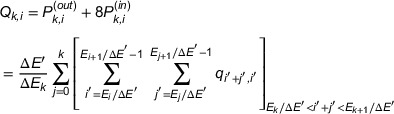




For a uniform irradiation the number of input events at a given energy bin k is the same for all the pixels ($n_{k}^{\left(t_{r}\right)}=n_{k}^{\left(c\right)}=n_{k}$). Following the same arguments of Section 2.2.2, the number of raw counts 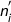 for a generic acquisition with a limited number of energy bins can be expressed as a function of the input number of events $n_{i}$ and of the splitting probabilities $Q_{k,i}$ as:

(18)

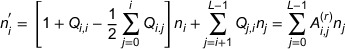




Equation (18) defines the elements of a new L×L response matrix $A^{\left(r\right)}$, adapted to the wider energy bins of the multi‐comparator system with a limited number of energy thresholds. Equation (18) has the same form of Equation (12) and can be solved to retrieve the number of input events $n_{i}$ with the same method described in Section 2.2.2.

#### 2.2.4 Nonuniform irradiation

In the previous sections the splitting probabilities, determined from the number of coincidences and applied to correct the CS spectral distortions in uniform irradiations, are a combination of CS‐IN and CS‐OUT transition probabilities (Equation (8)), which do not depend on the spectrum and on the spatial distribution of counts. In case of different input spectra in each pixel, the effect of CS on the number of counts is given by Equation (1), which solution requires the separation of the two transition probabilities. We propose two preliminary methods to unfold the spectral distribution of counts in each pixel.

In the first method (named CBRM‐IMG1) the number of input events in the nearby pixels which contribute to the CS‐IN transitions are assumed to coincide with the number of input events in the reference pixel ($n_{k}^{\left(t_{r}\right)}=n_{k}^{\left(c\right)}$). In this case the number of raw counts in each pixel is given by Equation (12) (or Equation (18) for a reduced matrix), that can be solved separately for each pixel to determine the number of input events. A spatial blurring is expected with this method, due to the assumption of an equal number of events for all the pixels of the 3x3 block centered to each reference pixel.

The second method (named CBRM‐IMG2) is based on the disentanglement of the CS‐IN and CS‐OUT transition probabilities. In particular, it is assumed that in events with CS most of the initial energy is still collected by the pixel where the original interaction occurs, thus neglecting one of the two transition probabilities in Equation (8). This assumption is reasonable when the CS events is due to the sharing of the signal between adjacent pixels, but it could not hold for events with emission and reabsorption of secondary photons. According to this assumption, the CS‐IN and CS‐OUT transition probabilities are:

(19) $$p_{k,i}^{\left(out\right)}=\left\{\begin{array}{cc} q_{k,i}\; & \text{for}\;i>k-i\\ 0\; & \text{for}\;i<k-i \end{array}\right.$$

(20) $$p_{k,i}^{\left(in\right)}=\left\{\begin{array}{cc} 0\; & \text{for}\;i>k-i\\ q_{k,i}\; & \text{for}\;i<k-i \end{array}\right.$$

When i=k−i, it is possible to use the relations given by Equation (4) to separate the CS‐IN and CS‐OUT contributions. It can be demonstrated that the transition probabilities for i=k−i are given by:

(21) $$\begin{array}{cc} p_{k,k/2}^{\left(out\right)} & =\frac{1}{2}\sum_{j=0}^{k}q_{k,j}-\sum_{j=k/2+1}^{k}q_{k,j}\\ p_{k,k/2}^{\left(in\right)} & =\frac{1}{8}\left[\frac{1}{2}\sum_{j=0}^{k}q_{k,j}-\sum_{j=0}^{k/2-1}q_{k,j}\right]. \end{array}$$

The number of raw counts $n_{i}^{{\left(c\right)}^{\prime }}$ in a given pixel is given by Equation (1) in terms of the number of input events in the same pixel $n_{i}^{\left(c\right)}$ and in the eight neighbouring $n_{i}^{\left(t_{r}\right)}$. This equation can be solved iteratively. In particular the number of input events at bin i of a given pixel at a step p of the iterative procedure is given by:

(22) $$\begin{array}{cc} & {\left\{n_{i}^{\left(c\right)}\right\}}_{p}\\ & =\frac{n_{i}^{{\left(c\right)}^{\prime }}-\sum_{k=i+1}^{L-1}p_{k,i}^{\left(out\right)}{\left\{n_{k}^{\left(c\right)}\right\}}_{p}-\sum_{k=i}^{L-1}p_{k,i}^{\left(in\right)}\sum_{r=1}^{8}{\left\{n_{k}^{\left(t_{r}\right)}\right\}}_{p-1}}{1+\sum_{j=0}^{i-1}p_{i,j}^{\left(out\right)}} \end{array}$$

where the number of input events in the adjacent pixels ${\left\{n_{k}^{\left(t_{r}\right)}\right\}}_{p-1}$ are determined in the previous step of the iteration. A small number of iterations (less than 10) are needed to converge to a stable solution.

It has to be remarked that the splitting probabilities $q_{k,i}$ used to define the transition probabilities in Equations (19), (20) and (21) can be adapted to arbitrary choices of energy bins with the method described in Section 2.2.3.

### 2.3 Monte Carlo simulation of a CdTe detector

The response of a pixelated CdTe detector of 1 mm thickness and segmented into 21x21 pixels was simulated with the Geant4 toolkit^32^ version 11.1.3. Different pixel sizes were considered, between 50 μm and 300 μm. X‐ray photons were generated with a direction perpendicular to the detector and an energy randomly selected from a polychromatic spectrum produced with the Spektr code.^33^ The photon source was placed at a fixed distance from the detector with transversal coordinates uniformly distributed to illuminate the whole detector or a portion of it.

The physics lists described in another study^34^ were used in Geant4 for an accurate simulation of emission and reabsorption of fluorescence and Compton photons. The method described in the same publication was followed to simulate energy sharing due to the electron charge cloud size. Each pixel is segmented in a matrix of 7x7 subpixels. For a photon impinging in a given sub‐pixel, an interaction point is randomly selected in the subpixel area with a uniform distribution. The released energy is therefore split into 500 equivalent parts, which are randomly distributed across the detector area according to a 2D isotropic Gaussian distribution centered at the interaction point. The energy in each pixel is the sum of the energy parts falling into the pixel area. In our simulation the standard deviation σ of the Gaussian spread depends on the initial size of the charge cloud $\sigma_{init}\left(E\right)$ and on the thermal diffusion of the cloud along the drift distance d:

(23) $$\sigma =\sqrt{\sigma_{init}{\left(E\right)}^{2}+\frac{2k_{B}TDd}{eV_{B}}}$$

In the previous equation $k_{B}$ is the Boltzmann constant, e the elementary charge, D the total sensor thickness, T the temperature (fixed at 300 K) and $V_{B}$ the bias voltage. In the simulation the incoming photons are assumed to enter the cathode side of the detector and the signal is collected at the anode. The initial cloud size $\sigma_{init}\left(E\right)$ depends on the energy E released by the photon interaction. The values of $\sigma_{init}$ used in our simulation are 0 μm, 2 μm and 8 μm for released energies of 0 keV, 35 keV and 100 keV, the same used in another study.^35^ The initial charge size for arbitrary values of the released energy E is determined from a second order polynomial fit of these values. Equation (23) does not include electrostatic repulsion during the drift, which is taken into account by using a bias voltage lower than the typical realistic values. In our simulation we set a value of $V_{b}=230$ V, which provides an average value of the total charge size of σ≃13 μm, relatively constant through the energy range between 1 keV and 120 keV explored in this study and compatible with the results from Koch‐Mehrin et al.^36^ for a 1 mm thick CdTe converter.

Several factors were not included in this simple simulation, like collection efficiency, signal formation, inter‐pixel gaps, passive materials. Electronic noise was simulated by adding a contribution to the energy of each detector pixel, randomly selected from a Gaussian distribution of mean zero and standard deviation $\sigma_{\mathrm{noise}}$. Pulse shape and PPU effects were not included in this study. It has to be remarked that this simple simulation was not intended to reproduce in details the real performance of a CdTe detection system, but to characterize the effects of CS mechanisms (including fluorescence and Compton emissions, reabsorption or escapes) for the validation of the statistical correction method described in Section 2.2. Actually, the CBRM correction is not based on the knowledge of the details of the detector response as it uses a matrix estimation based on the collection of coincidence counts to characterize all the contributions to CS effects.

For each input spectrum and detector condition, $10^{7}$ events were generated in the area of the detector illuminated by the beam. The events were divided into 10 data sets, and the analysis was repeated independently for each noise realization to evaluate the statistical fluctuations of the results. To determine the response matrix, the pixels at the edges of the detector were excluded and each of the other pixels was correlated with the sum of the energies of its eight neighbours. To test the reconstruction for nonuniform beams, a strong contrast image was simulated with a uniform irradiation limited to the central 7×7 pixels.

### 2.4 Data acquisitions with a Timepix4 hybrid detector

The experimental validation of the proposed method was performed by analysing data collected by a hybrid detector consisting of a 300 μm thick p‐on‐n silicon sensor with pixels of 55 μm pitch, bound‐bonded to a Timepix4 Application‐Specific Integrated Circuit (ASIC). The Timepix4 ASIC^31^, ^37^ was developed by the Medipix collaboration and features 448×512 channels with individual charge amplifiers and thresholds programmable at pixel level with local DACs. The total sensitive area is 24.64 × 28.16 ${\mathrm{mm}}^{2}$. A data‐driven readout modality provides, for each pixel whose signal crosses the threshold, the time of arrival (ToA) and the time the signal exceeds the threshold (Time‐over‐Threshold, ToT). The data were transmitted through two 2.56 Gbps links to an external readout system and saved on file. In the offline analysis the ToA was employed to identify clusters of simultaneous hits in neighbouring pixels while the ToT allowed, after proper calibration, to determine the energy deposited in each pixel. The availability of a timestamp for each pixel makes it possible to correlate the energy information of coincident hits and validate the CBRM method. It has to be underlined that, even if the offline analysis was performed on data lists containing information for each pixel hit, the correction method of the present study is intended to be applied to the integral number of counts stored internally in a standard multi‐discriminator chip, with obvious advantages in terms of hardware simplicity and readout speed.

Monochromatic X‐ray beams with 18 energies between 8.5 keV and 40 keV were produced at the SYRMEP beamline of the Elettra Synchroton in Trieste, Italy.^38^ Details on the experimental conditions and on the calibration procedures followed to equalize the thresholds and convert the ToT measurements into energy values were described in a previous studies.^39^ The SYRMEP beam cross‐section, defined by tungsten slits, was 5.2 mm (vertical) × 28.6 mm (horizontal) with a vertical Gaussian profile, covering 48k detector pixels. The detector was moved vertically during the acquisition to illuminate all the pixels.

Acquisitions with polychromatic spectra were performed using a microfocus X‐ray tube with a W anode operated at 50 kV and 0.1 mA. The CBRM matrix was determined with a flat‐field acquisition and afterwards applied to correct the spectrum attenuated by a solution of an Ag contrast agent. For these data sets, the detector calibration was based on test‐pulses and on the energies of the K‐edges of different contrast agents (Ag, Ba, I). The analysis was restricted to a region‐of‐interest (ROI) of 1500 pixels, corresponding to the central part of the vial containing the Ag solution.

The noise discrimination thresholds were set to 1000 $e^{-}$ (3.6 keV) for the SYRMEP acquisition, and to 300 $e^{-}$ (1.1 keV) for the X‐ray tube acquisition, both well above the Timepix4 typical equivalent noise charge (ENC) of 70 $e^{-}$ rms.^37^ In both tests the detector was operated at a bias voltage of 100 V.

The statistics collected at SYRMEP consisted in a number between 18 and 40 million of pixel hits for each beam energy while, for the polychromatic acquisitions, 10M hits were collected for the calibration run and 4M for the Ag sample within the ROI. The data sets were divided into 10 parts which were analysed independently for the evaluation of the statistical errors.

Pixels hits with ToA values within an interval of 2 μs were grouped to form an event. The mean number of pixel hits per event was 2.5 and 1.5 respectively in the SYRMEP and X‐ray tube acquisitions. Considering the area covered by the beam or by the selected ROI, the probability that multiple hits in the same or adjacent pixels were produced by independent incident photons was estimated to be less than 1 per mill at SYRMEP and less than 1% for the acquisitions with the X‐ray tube. Therefore, PPU effects are negligible for all the acquisitions.

### 2.5 Validation of the CBRM correction

In the simulation studies, the CBRM corrections were compared with reference distributions obtained by summing for each event the energies of all the pixels without the addition of noise. The reference distributions were also compared with the results of a C8P1 ACS algorithm.^23^ In the C8P1 algorithm each pixel is connected to four summing nodes, each including a group of 2×2 pixels. A set of comparators and discriminators is used to assign to the pixel with a signal higher than all its neighbours the analog sum of the node with the highest signal value, if this sum is above a minimum threshold. In the simulation, different noise discrimination thresholds are applied to the output of each pixel ($thr_{\mathrm{pxl}}$), to the sum of the eight pixels used in the calibration ($thr_{\mathrm{sum}}$) and to the analog sum of the C8P1 algorithm ($thr_{\mathrm{ACS}}$).

For the experimental tests, the response matrix was determined by using the list of hits recorded after a noise discrimination threshold applied to the single pixels in the acquisition stage. The CBRM corrections were compared with the results of a clustering algorithm based on 3×3 summing nodes. This clustering algorithm assigns to each pixel the sum of its energy and of the eight surrounding pixels and selects the clusters with the maximum value of the sum. One or more non‐overlapping 3×3 clusters are identified within the same time window and, for each selected cluster, the energy sum is assigned to the pixel with maximum individual energy. The minimum threshold applied during the acquisition was well above the noise rms and therefore no other noise thresholds were applied to the sums used in the calibration phase and in the clustering algorithm.

The agreement between different spectra was quantified in terms of mean absolute percentage error (MAPE), defined as:

(24) $$MAPE=\frac{100}{N}\sum_{i=0}^{L-1}\frac{|n_{i}^{corr}-n_{i}|}{n_{i}}$$

where L is the number of energy bins, $n_{i}^{corr}$ the number of counts in one pixel at energy bin i after correction with the CBRM method or obtained by the ACS algorithm, and $n_{i}$ the number of events in the same pixel at energy bin i for the reference spectrum. Bins with a number of counts lower than 10% of the average number of bin counts were excluded from the sum to limit the sensitivity of the MAPE error to bins with high statistical fluctuations. The factor N in Equation (24) is the number of bins included in the sum.

For monochromatic spectra, the mean values and the standard deviations obtained from Gaussian fits to the peaks of the count distributions were evaluated.

## 3 RESULTS

### 3.1 Simulation studies

The response of a CdTe detector to a polychromatic spectrum from an X‐ray tube with W target and 1.6 mm Al filter operated at 120 kV was used to determine the elements of the detector response matrix. A detector with pixel size 200 μm was simulated, a random noise with $\sigma_{\mathrm{noise}}=1$ keV was added to the raw energy of each pixel, the width of each energy bin was set to 1 keV. The noise discrimination threshold for each pixel was fixed to the minimum level to reject fake counts due to noise ($thr_{\mathrm{pxl}}=5$ keV), while the minimum thresholds applied on the energy sums employed for the CBRM matrix determination and by the ACS algorithm were $thr_{\mathrm{sum}}=3$ keV and $thr_{\mathrm{ACS}}=10$ keV, respectively. The motivation of these choices will be justified in the following.

**FIGURE 3:**
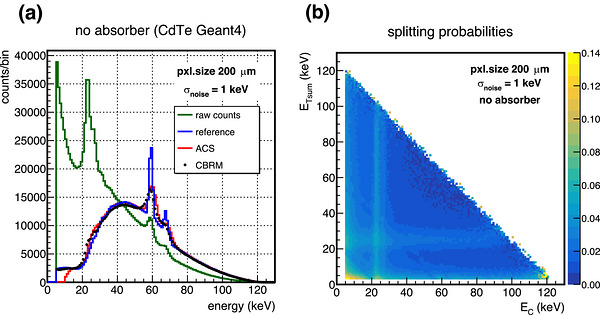
(a) Raw count distribution (green histogram), ideal count distribution (blue), result of an ACS algorithm (red) and of the CBRM matrix correction (black markers) for a polychromatic spectrum from an X‐ray tube at 120 kV on a CdTe detector of 1 mm thickness and 200 μm pixel size with $\sigma_{noise}=1$ keV (Geant4 simulation). The noise discrimination thresholds were $thr_{pix}=5$ keV, $thr_{sum}=3$ keV, $thr_{ACS}=10$ keV. The CBRM correction was based on a response matrix determined with the same data. (b) Splitting probabilities determined from the raw counts and coincidences for the same spectrum as a function of the residual energy in the reference pixel ($E_{C}$) and in its eight surrounding pixels $(E_{Tsum}$).

Figure 3a shows the ideal count distribution as a blue histogram, the raw counts as a green histogram, the results of the ACS algorithm in red, while the black markers correspond to the counts in each energy bin corrected by solving Equation (11). Here and in the following figures the error bars could be hidden by the line width or marker size. The ACS and CBRM methods compensate reasonably well the CS spectrum distortions, excluding the lower energy region for the ACS algorithm (due to the higher minimum threshold) and the region around the fluorescence peak at 23 keV. Both the correction methods are indeed based on charge summing or coincidences between a limited number of pixels and are not able to compensate for the fraction of fluorescence photons emitted and reabsorbed in two pixels further away. The comparison of the restored count distributions with the reference gives MAPE values of 7.4% (CBRM) and 11.8% (ACS). The counting efficiency (defined as the ratio of the total number of reconstructed counts over the number of input events) are 99.3% (CBRM) and 99.9% (ACS).

Figure 3b shows the splitting probabilities $q_{k,i}$ determined with Equation (8) using the coincidence counts obtained from the spectrum of Figure 3a and displayed as a function of the residual energy in the reference pixel (bin i and mean energy 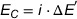) and in the eight surrounding pixels (bin k−i corresponding to a mean energy 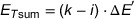). The splitting probabilities are particularly high at low energies, where CS effects are more severe, and in the presence of fluorescence photons (horizontal and vertical bands at 23 keV). It is worth noting that the width of the energy bins used to define the response matrix are comparable with the noise rms; this produces a blurring of the matrix contents but does not significantly affect the restoring capabilities (the MAPE from the comparison of the CBRM corrected counts with the ideal spectrum for a noiseless detector is only slightly reduced from 7.4% to 5.8%).

**FIGURE 4:**
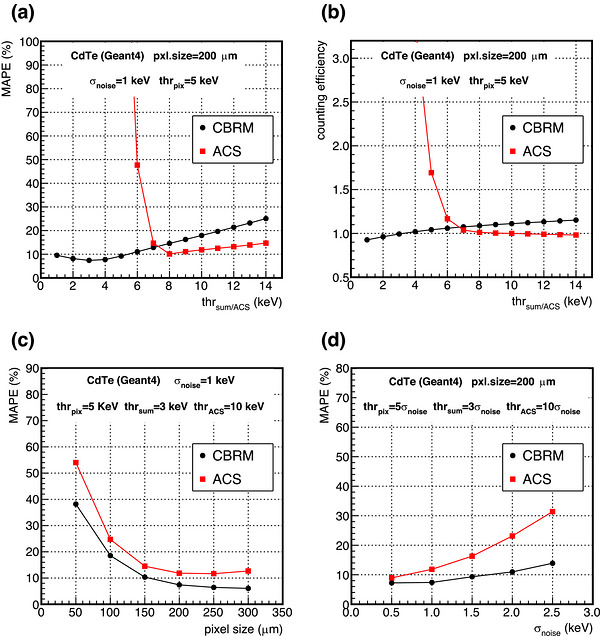
(a) MAPE values as a function of $thr_{\mathrm{sum}}$ (CBRM method, black markers) and $thr_{ACS}$ (ACS method, red markers) for the corrections of a 120 kV polycromatic spectrum and a CdTe detector (pixel side of 200 μm, $\sigma_{\mathrm{noise}}=1$ keV, $thr_{\mathrm{pxl}}=5$ keV). (b) Counting efficiency (total number of reconstructed counts over the number of input events) as a function of $thr_{\mathrm{sum}}$ (CBRM) and $thr_{\mathrm{ACS}}$ (ACS). (c) MAPE values of the CBRM and ACS reconstructed spectra as a function of the pixel size. (d) MAPE values for the CBRM and ACS reconstructed spectra as a function of $\sigma_{\mathrm{noise}}$ (pixel size 200 μm, noise discrimination thresholds proportional to $\sigma_{\mathrm{noise}}$).

The performance of the ACS and CBRM corrections depends on the pixel size, on the noise level and on the choice of the noise discrimination thresholds. Figure 4a shows the MAPE values from the comparison of the CBRM (black markers) and ACS (red markers) count distributions with the reference one as a function of the minimum thresholds $thr_{\mathrm{sum}}$ and $thr_{\mathrm{ACS}}$, obtained with the same spectrum, $\sigma_{\mathrm{noise}}$ and $thr_{\mathrm{pxl}}$ employed for the simulation of Figure 3. Figure 4b shows the counting efficiency in the same conditions as a function of the two thresholds. The ACS count distributions are severely affected at low $thr_{ACS}$ thresholds. This is caused by fake counts from summing nodes signals exceeding the noise discrimination threshold due to the sum in quadrature of the noise contributions from four pixels. At the opposite, the CBRM method is not affected by the additive noise in $T_{\mathrm{sum}}$, because the analog sum of eight pixels is used in coincidence with a hit in the reference pixel above $thr_{pxl}$. From Figure 4b it also appears that the counting efficiency for the CBRM method depends on the minimum threshold $thr_{\mathrm{sum}}$ used in the calibration phase. In particular, the CBRM method tends to overestimate the number of counts at higher value of $thr_{\mathrm{sum}}$ due to the lack of information on the elements of the response matrix at low energies. The best results for the CBRM reconstruction are obtained by setting a noise discrimination threshold for $T_{\mathrm{sum}}$ lower than the minimum threshold $thr_{\mathrm{pxl}}$ applied to single pixels. With a noise level $\sigma_{\mathrm{noise}}=1$ keV the nominal threshold values used in all our simulations are the ones applied in the reconstruction of Figure 3 ($thr_{\mathrm{pxl}}=5$ keV, $thr_{\mathrm{sum}}=3$ keV and $thr_{\mathrm{ACS}}=10$ keV).

Figure 4c shows the MAPE values for the ACS and CBRM methods as a function of the pixel size. The two methods provide similar results, with a degradation of the reconstructed spectrum with respect to the reference one for pixels of 100 μm or less, where the assumption of a cluster of hits contained in a limited number of pixels, on which both the ACS and CBRM methods rely, does not hold anymore. The higher MAPE values for ACS are due to the higher noise discrimination threshold which prevents the reconstruction of the lower energy part of the spectrum.

In Figure 4d the MAPE values for the CBRM and ACS spectral corrections are shown as a function of the noise level $\sigma_{\mathrm{noise}}$ for a pixel size of 200 μm. The minimum thresholds on single pixels and on the analog sums of the ACS and CBRM methods are scaled with the noise rms as $thr_{pxl}=5\cdot \sigma_{\mathrm{noise}}$, $thr_{\mathrm{sum}}=3\cdot \sigma_{\mathrm{noise}}$ and $thr_{\mathrm{ACS}}=10\cdot \sigma_{\mathrm{noise}}$. The performance of both the methods worsens with the increase of the electronic noise, in particular for the missing counts at the low energy part of the spectrum at higher minimum threshold values for the ACS method.

**FIGURE 5:**
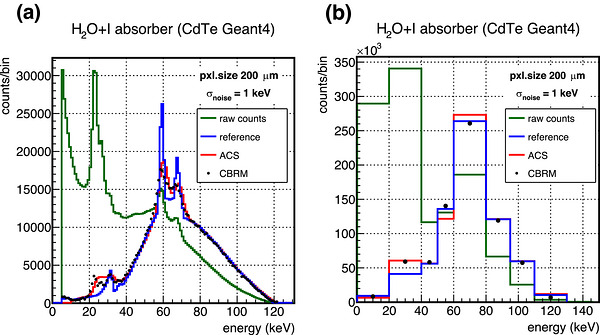
Raw count distribution (green histogram), ideal count distribution without CS (blue histogram), results from an ACS algorithm (red histogram) and CBRM correction (black markers) for an X‐ray spectrum of a tube at 120 kV attenuated by 120 mm of $H_{2}O$ and 0.2 mm of Iodine. A CdTe detector of 1 mm thickness and 200 μm pixel size with 1 keV rms noise was simulated with Geant4. In (a) the CBRM correction used the same energy bins of the response matrix determined with data collected without attenuation. In (b) the same matrix was adapted to a lower number of energy bins of different widths.

The response matrix determined with the simulation of Figure 3 was applied to reconstruct the 120 kV spectrum attenuated by 100 mm of $H_{2}O$ and a 0.2 mm of Iodine, using the same pixel size (200 μm) and the nominal settings for the electronic noise and the minimum discrimination thresholds. The results from the solution of Equation (12), for an acquisition with the same energy bins employed for the determination of the response matrix, are shown in Figure 5a. In these conditions the MAPE values from the comparison of the corrections with the reference count distribution are 20.0% (CBRM) and 22.8% (ACS), and the counting efficiencies are 101.7%(CBRM) and 102.0%(ACS). Slightly worse results were obtained if the CBRM correction was performed with a matrix determined with the count and coincidences collected with the same attenuated input (MAPE = 22.8%). These observations demonstrate the possibility to apply the response matrix determined with a calibration acquisition of a polychromatic beam covering the whole energy range under investigation to data acquired with other input spectra attenuated by materials placed between the source and the detector.

Figure 5b shows the same spectrum reconstructed applying the method described in Section 2.2.3 to adapt the response matrix to a lower number of energy bins of different widths. The MAPE values from the comparison of the CBRM and ACS restored spectra with the reference count distribution are 12.7% and 13.4% respectively. The counting efficiencies are 102.3% (CBRM) and 101.9%(ACS). It has to be mentioned that in general the performance of the CBRM reconstruction worsens by reducing the number of energy bins for the matrix determination, while a matrix determined with a fine energy division and the use of a rescaled matrix allows to provides a good spectrum restoration comparable with the results of the ACS algorithm independently of the number of energy bins used for the acquisition.

**FIGURE 6:**
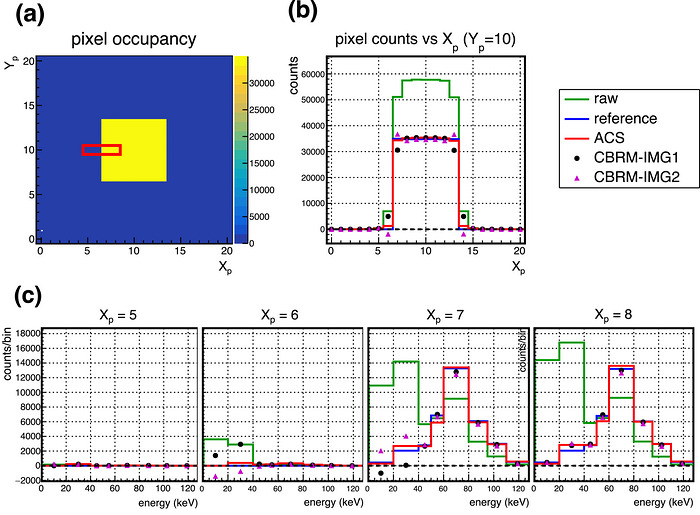
(a) Number of input events in the detector pixels for an irradiation of the central 7×7 pixels with a 120 kV X‐ray beam attenuated by a solution of $H_{2}O$ and iodine (CdTe Geant4 simulation, pixel size 200 μm). (b) Number of input events (blue histogram), raw (green histogram) and reconstructed counts (red histogram for ACS, black markers fo CBRM‐IMG1, magenta markers for CBRM‐IMG2) as a function of the pixel position $X_{p}$ for a row of pixels with $Y_{p}=10$. (c) Energy distribution of the number of input events and counts in the four pixels highlighted in (a).

A preliminary test of a CBRM reconstruction with a nonuniform irradiation was performed by illuminating only the central 7×7 pixels of the detector with a 120 kV X‐ray beam attenuated by 100 mm of $H_{2}O$ and a 0.2 mm of Iodine. The simulation was performed with a pixel size of 200 μm, the nominal noise and minimum discrimination thresholds, and the response matrix of Figure 3 was adapted to match the energy bins of Figure 5b. The number of input events for each detector pixels are shown in Figure 6a as a function of the pixel indices $X_{p}$ and $Y_{p}$. The CS correction was performed with the two methods described in Section 2.2.4 to retrieve the number and the energy distribution of input events in individual pixels, which were compared with the reference and with the result of the C8P1 algorithm. Figure 6b shows the total number of input events (blue), of raw (green) and reconstructed counts for all the pixels in a row with $Y_{p}$ = 10. The red histograms corresponds to the result of the ACS algorithm, the black and magenta markers to the reconstructed number of counts for the CBRM‐IMG1 and CBRM‐IMG2 methods, respectively. For the ACS algorithm the transition of the number of counts at the edge of the illuminated area is very sharp, confirming the good performance of online charge summing techniques in high contrast imaging applications. The transition is softer for the CBRM‐IMG1 method, as expected since this reconstruction method applied to each pixel does not use information on the counts of its adjacent pixels. The CBRM‐IMG2 reconstruction provides a shaper transition on the number of reconstructed counts, but with ringing artifacts with over‐ or under‐estimations of the total number of counts for the pixels in close proximity of the edge. Figure 6c shows the energy distribution of the number of input events (blue histogram), raw (green) and reconstructed counts (red histogram for ACS, black markers for CBRM‐IMG1, pink markers for CBRM‐IMG2) for each of the four pixels highlighted by the red box in Figure 6a. At the center of the illuminated area both the CBRM‐IMG1 and CBRM‐IMG2 methods provide the same spectral reconstruction already shown in Figure 5b, comparable with the ACS reconstruction. For the pixels in close proximity of the edge, the number of counts in the lowest energy bins are not estimated correctly by the CBRM methods. The CBRM‐IMG2 method provides results closer to the reference or to ACS distributions than the CBRM‐IMG1 method, but with negative counts in the pixel closest to the illuminated area. The artifact of the CBRM‐IMG2 reconstruction at low energy and close to the edge of the image are due to the production and reabsorption of fluorescent photons, which invalidates the hypotheses made for the model.

### 3.2 Study of experimental data

The analysis of the data collected at the SYRMEP beamline with the Timepix4 silicon detector was performed using energy bins of 0.5 keV width. The response matrix was determined by merging all the data collected at 18 different energies to define the splitting probabilities over a wide energy range. The CBRM matrix was therefore applied to recover the spectrum for each single energy by solving Equation (12) and the results were compared with the spectra obtained from a clustering algorithm based on 3x3 pixel blocks (Section 2.5).

**FIGURE 7:**
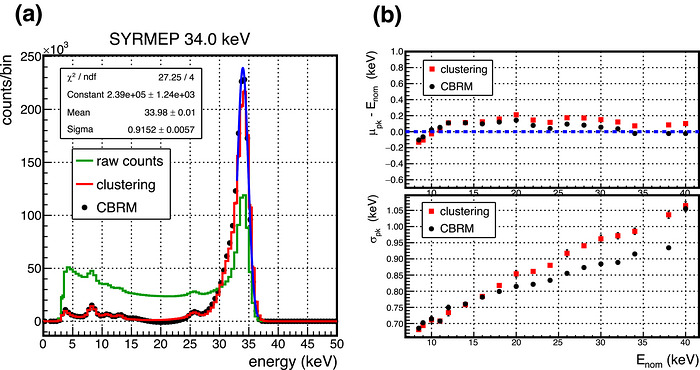
(a) Pulse‐height distribution of raw counts (green), of counts from a 3×3 clustering algorithm (red), and from the CBRM correction (black marker) for monochromatic 34 keV X‐rays (SYRMEP beamline at the Elettra synchrotron) acquired with a 300 μm thick SI detector readout by a Timepix4 ASIC. The result of a Gaussian fit to the peak is shown in the insert on the figure and as a blue curve. (b) Mean values (top) and standard deviations (bottom) from Gaussian fits of the peaks of the count distributions of SYRMEP monochromatic beams as a function of the nominal X‐ray energy for the clustering (red markers) and CBRM correction (black markers).

Figure 7a shows the count distributions for an irradiation with monochromatic 34 keV X‐rays: the green histogram corresponds to the raw counts, the red to the count distribution from the clustering algorithm, the black markers to the CBRM correction. The MAPE from the comparison of the CBRM correction with the clustering is 23.1%. A Gaussian fit to the peak of the CBRM distributions (excluding the tail at lower energy) is also shown in figure as a blue curve. At this energy the total number of counts from the CBRM correction is 3.2% higher than the number of clusters. The excess of counts is due to the use of eight pixel hits each above the minimum noise cut for the definition of the energy sum used in the matrix determination. This results in an effective high minimum discrimination threshold for $T_{\mathrm{sum}}$ and consequent missing informations in the low energy part of the response matrix.

Gaussian fits to the peaks of the clustering and CBRM distributions were performed separately for each of the 18 energies between 8.5 and 40 keV used in the acquisitions. Figure 7b shows on the top the difference between the mean values of the Gaussian fit $\left(\mu_{pk}\right)$ and the nominal energy $E_{\mathrm{nom}}$ as a function of the nominal energy for the clustering (red markers) and the CBRM correction (black markers), on the bottom the standard deviations $\sigma_{pk}$ from the fits. The CBRM reproduces well the results from the clustering algorithm, with typical MAPE values from the comparison of the two distributions between 10% at low energies and 30% at higher energies. The discrepancies are mainly due to an offset of about 0.1 keV between the peak positions of the two distributions for $E_{\mathrm{nom}}$ greater than 20 keV and to an excess of counts from the CBRM correction up to 3% at the highest energies. Excluding the lower energies and the anomalous value at 40 keV, the standard deviations for the CBRM corrections are smaller than those obtained after clustering.

**FIGURE 8:**
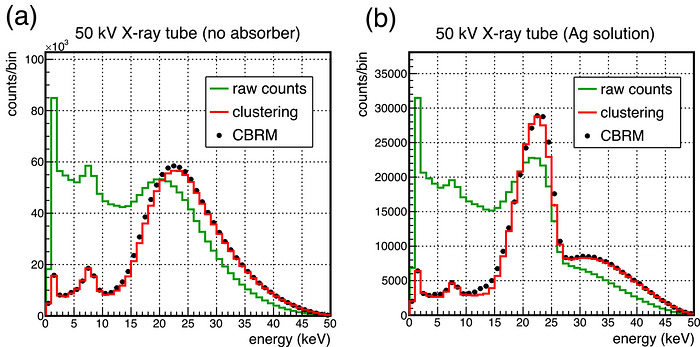
Pulse‐height distribution of raw counts (green), of counts from a 3x3 clustering algorithm (red), and from the CBRM correction (black marker) for an acquisitions of X‐rays from a 50 kV tube without absorber (a) and with the beam attenuated by an Ag solution (b). A ROI of 1500 pixels was selected and the CBRM correction was based on the matrix determined with the spectrum without absorber.

For the acquisitions with a polychromatic 50 kV X‐ray beam, the response matrix was determined with a flat‐field illumination without absorber and applied to restore the count distribution of a spectrum attenuated by an Ag solution. The count distributions for the two spectra are reported in Figure 8, respectively for the acquisition without absorber (a) and with the absorber (b). The green histograms correspond to the raw counts, the red to the results from a 3×3 clustering algorithm, the black markers to the counts corrected with the CBRM. The MAPE error from the comparison between the clustering and corrected distributions are 3.6% (without absorber) and 6.2% (with absorber). In both cases the CBRM correction provides a total number of counts 3.4% greater than the number of reconstructed clusters. The CBRM matrix determined with a reference spectrum provides a restored spectrum similar to that from a clustering algorithm. The rapid decrease at the Ag K‐edge of 25.5 keV is clearly visible in both reconstructed spectra shown in Figure 8b. No attempt was done in this study to quantify the concentration of Ag in the solution with a K‐edge analysis. However, the good comparison with the performance of a standard clustering algorithm indicates that the CBRM reconstruction can restore the CS spectral distortion and could be applied for contrast agent identification and quantitative material discrimination.

## 4 DISCUSSION

The studies based on simulations and on X‐ray data collected with a Timepix4 hybrid silicon detector showed the effectiveness of the CBRM method to restore the spectral distortions due to CS effects. In the simulation studies the spectra restored with the CBRM and a standard ACS algorithm were compared with the output of an ideal detector of infinite pixel size. For the experimental data, an offline 3x3 clustering algorithm was used for comparison. In both cases the results from the CBRM corrections were comparable to those obtained by analog summing or offline clustering. It was also demonstrated that the application of the CBRM matrix is independent of the acquired spectra.

The ACS algorithm requires a higher noise discrimination threshold than the CBRM method, due to the contribution of several pixels to the noise of the energy sums used by the ACS. A lower noise discrimination threshold is tollerated and preferable for the energy sum of the eight pixels $T_{\mathrm{sum}}$ used for the CBRM matrix determination, since this sum is used in coincidence with the signal above the noise cut of one pixel. The increase of the noise discrimination threshold on $T_{\mathrm{sum}}$ would result in the missing reconstruction of the low energy elements of the CBRM matrix and an over‐estimation of the total number of corrected counts. The performance of both CBRM and ACS algorithms corrections degrades for small pixel size, when CS involves clusters with more than 2×2 pixels.

The CBRM method requires the determination of the response matrix for a large number of energy bins of small width. In the acquisition scheme of Figure 1 the number of bins depends only on the settings of two DAC converters which levels can be changed in small steps. However, the number of thresholds used in standard multi‐discriminator electronics is in general lower than the number of thresholds needed for an accurate determination of the response matrix. It was shown that it is possible to rescale the response matrix to match the energy bins of a standard acquisition system. The correction in such a case is almost insensitive to the number of bins employed by the standard pulse‐height acquisition electronics and is more accurate than using a response matrix defined with the same energy bins of the standard acquisitions.

The performance of the method with nonuniform irradiations was investigated with a simulation of a high contrast image obtained by illuminating only the central part of the detector. Some assumptions were made in the algorithms used for the spectrum reconstruction of each pixel, which however do not affect the counting and spectral restoration, with the exception of the two pixels in close proximity of the edge of the irradiated region where some artifacts appear at low energy, due to the emission and reabsorbtion of fluorescent photons. Futher studies will be performed in the future to quantify and optimize the performance of the image reconstruction with the CBRM method.

The method relies on a uniform behaviour of the detector pixels. It was implicitly assumed that in a realistic multi‐discriminator system the thresholds are programmable at pixel level, and possible variations in the energy response can be compensated after a proper calibration of the detector.

The Timepix4 ASIC used for the experimental validation was not designed for high X‐ray fluxes typical of medical applications. However, the distortions due to CS effects and the statistical algorithms employed by the CBRM method do not depend on the count rate or the system dead‐time. Therefore, the validation performed with the high dead‐time Timepix4 hybrid detector at low rates can be extended to detectors with lower dead‐times operating at high X‐ray fluxes. In Timepix4, the pixel energy is measured with a Time‐over‐Threshold method rather than a multi‐discriminator system. The experimental validation remains valid because the CBRM method is independent of how the pixel energy is determined.

There are some limitations in the present study. The simulation neglects charge trapping, polarization, inter‐pixel gaps and other effects. However, the method does not rely on an accurate knowledge of the detector response and CS effects, as all the relevant information needed to restore the spectral information are determined by the coincidence counts collected in the calibration phase.

This study also neglects PPU effects at high rates which could mimic correlated counts in two neighbouring pixels providing a wrong prediction of the response matrix. The problem could be limited in the calibration phase by using low beam currents. However, in a subsequent high‐rate acquisition, especially for clinical imaging, PPU effects are not negligible and produce additional spectrum distortions. PPU distortions were absent or negligible in the present study. In the future the impact of PPU on the CBRM reconstruction and possible strategies for a combined mitigation of PPU and CS effects will be explored.

There are already many proposals to mitigate CS and/or PPU distortions with a post‐processing of the recorded counts, but they are based on physics models, simulations or data fitting which are specific for a particular detector or acquisition setting and in general are not applicable in a generic situation. Model‐independent methods proposed for CS mitigation are based on dedicated readout electronics, like anticoincidence circuits,^40^ ACS techniques, and multi‐energy inter‐pixel coincidence counters (MEICC).^28^ The implementation of these solutions is more complex than CBRM, requiring multiple inter‐pixel charge summing and communications or many coincidence gates for MEICC. Instead, our proposal requires only a simple AND logic between one pixel and the sum of the signals of the eight neighbours to determine the response function in a calibration stage. The calibration can be performed with a dedicated small detector with the same sensor employed for subsequent imaging acquisition or implementing the coincidence readout in a small part of the full‐size detector. The following acquisitions are performed with standard multiple discriminators and counting electronics, with no modification to the existing X‐ray PCDs. In both the MEICC method and the proposed CBRM method, no additional dead‐time is added during the acquisition, allowing higher acquisition rates with respect to readouts with ACS circuits.

## 5 CONCLUSION

A method is proposed for the offline compensation of charge sharing spectral distortions in photon counting X‐ray acquisitions. The method is based on the characterization of CS effects for a particular detector through a response matrix (named CBRM) whose elements are determined by counting the coincidences between the signals from one pixel and from the analog sum of its eight neighbours for different combinations of energy bins in a uniform irradiation with a polychromatic spectrum. Once the CBRM matrix is determined with a fine division of the energy range, it can be applied to restore CS distortions of other spectra using as input only the counts for different thresholds of a conventional multi‐discriminator readout electronics with a lower number of energy bins.

The CBRM method was assessed with a Monte Carlo simulation of a spectrum from a 120 kV X‐ray tube on a CdTe detector. The CBRM corrections were compared with reference spectra of an ideal detector with infinite pixel size and with the results of an analog charge summing technique. The CBRM method provides results similar to those of the ACS algorithm but with lower noise discrimination thresholds, thus allowing to extend the spectral correction to lower energies. A preliminary reconstruction of the spectral distribution in a nonuniform irradiation with high contrast provides good results, but with some artifacts at low energy in the pixels close to the irradiation edge with respect to the ACS algorithm.

The experimental validation of the method was performed with data of monochromatic and polychromatic X‐ray beams collected with a hybrid silicon detector readout by the Timepix4 electronics. The CBRM corrections were compared with an offline clustering algorithm, providing similar results in restoring the spectra. Gaussian fits of the reconstructed peaks of monochromatic spectra from the two corrections provide comparable mean and standard deviation values.

In conclusion, the proposed method is independent of physics models or parameterizations, is simple to realize, as the required coincidence electronics involve only a block of nine pixels in a calibration run, and the response matrix can be applied to correct different spectra collected by a standard photon counting detector with pulse‐height capability without introducing additional dead‐times. Spectrum distortions due to pulse pileup were not investigated in this study.

## CONFLICT OF INTEREST STATEMENT

The authors declare no conflicts of interest.
